# On the Application of Cotton to Burns

**Published:** 1813-02

**Authors:** 


### *To the Editors of the Medical and Physical Journal.*

GENTLEMEN,

HAVING read, in several American Newspapers, of the successful treatment of burns and scalds, by the simple application of *Cotton*, I am induced to forward to you part of a Newspaper containing remarks thereon.

The

The remedy, to me, appears so new, that, should you deem it of sufficient consequence, it may, in your hands, be more generally known through the medium of your periodical publication.

I am, Gentlemen,

With great respect,

Your very humble Servant,

H. C.

Fellow Med. Soc. Blenheim-street.

*H. M. Ship San D——.*

*Halifax Harbour, North America,*

*Nov.* 11, 1812.

---

*From the Baltimore Medical and Philosophical Lyceum.*

DALLAM *on the* USE *of* COTTON *in* BURNS.

DEAR SIR,

I received your favor of the 22d instant, and hasten to communicate to you the result of my inquiries and information respecting the accidental discovery of the beneficial effects produced by the application of *cotton* to *scalds and burns*. Should you think the facts stated worthy a place in the Lyceum, they are entirely at your disposal.

CASE I.—A child of Captain R. in this neighbourhood, aged five years, was standing alone before the kitchen fire, when a large kettle of *boiling water* fell, and was suddenly dashed over nearly its whole body. The affrighted mother, who was sitting in the adjoining room picking and carding cotton, flew to her darling child's assistance, and, having undressed it as quickly as possible, discovered it to be badly scalded. No medical aid being near, in the agitation and distress of her distracted mind she seized a large bundle of cotton, and applied it over the whole of the scalded parts. Soon after the application, the tortured and screaming infant became perfectly quiet, and fell into a gentle and easy slumber. The cotton was suffered to remain on several hours, and, when it was removed, there was not the least appearance of inflammation remaining. This case was communicated to me by a person of veracity, who saw the child soon after the accident, and there can be no doubt of its correctness.

CASE II.—Soon after the termination of the preceding cure, a black child, belonging to Mr. S. of this county (Harford), aged eighteen months, while playing on the hearth, fell into the fire and was badly burned. Its screams drew its mistress to the kitchen, and, having just before heard of the successful experiment with the cotton, she immediately covered the parts with a thick bolster of the same. She informed me that it succeeded completely, and that she had become become convinced of its efficacy, though before she had no faith in it.

Case III.—About three months ago, *my own little daughter* was crawling unobserved near the hearth, and overturned a tin cup of boiling water, which a servant had just before brought to make a lather for the purpose of shaving. The water (which was boiling) extended over the whole of one of her arms, and scalded it exceedingly. Being present, I immediately bound up the affected parts with fine cotton. She ceased crying in a few minutes, and was well enough to ride to Havre-de-Grace with her mother the same day. When the cotton was removed, the cuticle was scarcely discolored.

In what manner does cotton act in lessening pain and preventing or removing inflammation from scalds and burns? Is it by excluding atmospheric air simply, or by some specific power? Whatever theory may be adopted to explain the facts, they must remain undoubted, and if a still greater number would add to their validity, I could state them, for I have witnessed the same effects from the same application in many other cases. I presume those I have detailed will be sufficient to encourage others to give the remedy a fair trial.

I believe, moreover, that the application might be serviceable in other inflammations, if properly applied. The following case would seem to favor such an opinion:—A respectable lady of this county was seized with a violent pain in one of her jaws, which was supposed to have been rheumatic. The pain was excruciating, and the inflammation and swelling great. The usual remedies had been applied for several days, without affording the least relief. She was advised to try the external application of cotton to the inflamed part. It succeeded completely, and she assured me that in twelve hours after the application the inflammation and swelling entirely disappeared.

Accept, dear sir, of my best wishes for the success of the Lyceum, and believe me

Your's very sincerely,

*Harford County, Jan.* 20, 1811. William W. Dallam.

The simplicity of the application of cotton in obviating the action of fire upon the living fibre, and the want of success in the use of means much more plausible, left my mind in a state betwixt incredulity and approbation. Having sometimes failed in the treatment of inveterate burns by all the modes hitherto recommended, and having lately been disappointed in the effects to have been expected from the Spiritus Terebenthinæ (the celebrated remedy of Dr. Kentish), where gangrene ensued notwithstanding its immediate application, plication, I resolved to avail myself of the first opportunity of testifying the efficacy of the cotton. Only a few days had elapsed after the reception of Dr. Dallam's letter, before a case, in every respect adapted to my views, presented itself.

On the evening of the 7th day of February, while I was visiting the lady of Mr. John Leigh, of this city, the family was suddenly alarmed by the cries of William Wheatly, an adopted child of Mr. Leigh, aged thirteen months. He was immediately brought to Mrs. Leigh's room, where I was sitting, and upon examination I found that boiling water had been accidentally poured down his back from the spout of a tea-kettle. The water first came into contact with the cuticle between the scapulæ, opposite the second dorsal vertebra, and descended along the spine and an inch latterly to the right and left till it reached the glutæi muscles. In ten minutes after the accident, a covering of finely carded cotton was laid over the whole affected surface, and confined in close contact with the cuticle. As the cries of the child were expressive of the most excruciating pain, and the solicitude of the family for its repose great, I was questioned respecting the propriety of an anodyne? Having determined to rest the case solely upon the virtues of the cotton, I evaded the question, and resolved to remain with the patient, and observe as minutely as possible the progress of the case. In about fifteen minutes after the application, the child became less agitated; in half an hour ceased crying, and a profound sleep ensued in forty minutes from the accident. The child continued to sleep for about an hour, when he suddenly awoke, and, after crying a minute or two, became composed. The cotton was now removed, and another bolster applied. No mark of increased action was apparent, and a tranquil sleep soon followed. The child remained in this state, unmolested by pain or restlessness, till about six o'clock the next morning, when it awoke, resumed its usual habits of playfulness and good humor, took its usual morning repast, and discovered no symptoms of disease. Agreeably to my request, the cotton remained on the scalded parts till I removed it at nine o'clock. It was with difficulty I could trace even a rubefacience, although appearances had been so threatening the evening before. There was, however, a small exception to this pleasing appearance. There was a space of about an inch below the place first touched by the water that was vesicated. As I had not observed the precise situation of the cotton before it was removed, I was at a loss to account for this limited affection: I was informed, by one of the attendants, that the

 cotton cotton had slipped down during the night, and that the vesicated part had remained bare. The application was renewed as well to the vesication as to the other parts, and continued till the morning of the 9th, when it was finally removed. The vesication gradually declined, and, on the 12th, the cuticle peeled off without the least ulceration, or even a discharge. Although I cannot satisfactorily account for the operation of the cotton, I will state an occurrence that may lead to an experiment to determine it hereafter. Although the heat of the affected parts was augmented previous to the application, it was afterwards diminished to a degree less than the ordinary temperature of the skin, for, on the removal of the application, the parts were cooled, while the cotton was preternaturally heated. It was to have been expected that the preternatural heat of the skin would have been communicated to the cotton till an equilibrium would have been produced, according to the usual laws of caloric; but the fact was otherwise, for the cotton was heated in the same ratio that the skin was rendered cooler. If it were admissible to call to our aid the electric fluid so convenient and fashionable in cases of difficulty, we might hazard an hypothesis that cotton might be an excellent conductor, and possibly obey laws essentially different from those observed by common electricity, or the matter of heat.

EDITOR.

*Messrs. Websters and Skinners*—

A son of mine, a short time since, while playing, with other boys, with fire-balls, composed of tow dipped in spirits of turpentine, gunpowder, &c. was burned in a shocking manner, by having one of the fire-balls accidentally thrown in his face. Having noticed the application of cotton, recommended in the Baltimore Medical Lyceum, in cases of burns, I was induced to make the experiment. I covered the side of the face, which was now so much swollen as to close the eye, and blistered all over, with cotton: the skin also was in some parts broken, by his having washed it in cold water, immediately after the accident had taken place. I did not remove the cotton for ten days, during which time he never complained of the least pain; and when removed I found it perfectly well, leaving no sort of marks, except two or three spots rather darker than the rest of the skin, but which have since disappeared. As a remedy so efficacious, and so easily procured, ought to be made public for the benefit of our fellow-citizens, I request the liberty to communicate the same through the medium of your paper; and remain, your obedient servant, JOHN COOK.

*Albany, June* 23, 1812.

COLLEC-

